# Machine Learning and Smart Devices for Diabetes Management: Systematic Review

**DOI:** 10.3390/s22051843

**Published:** 2022-02-25

**Authors:** Mohammed Amine Makroum, Mehdi Adda, Abdenour Bouzouane, Hussein Ibrahim

**Affiliations:** 1 Département de Mathématiques, Informatique et Génie, Université du Québec à Rimouski (UQAR), 300 Allée des Ursulines, Rimouski, QC G5L 3A1, Canada; 2 Département d’Informatique et de Mathématique, Université du Québec à Chicoutimi (UQAC), 555 Boulevard de l’Université, Chicoutimi, QC G7H 2B1, Canada; abdenour.bouzouane@uqac.ca; 3 Institut Technologique de Maintenance Industrielle, 75 Rue de la Vérendrye, Sept-Iles, QC G4R 5B7, Canada; hussein.ibrahim@itmi.ca

**Keywords:** diabetes, wearables, digital health, glucose monitoring, artificial intelligence, machine learning

## Abstract

(1) Background: The use of smart devices to better manage diabetes has increased significantly in recent years. These technologies have been introduced in order to make life easier for patients with diabetes by allowing better control of the stability of blood sugar levels and anticipating the occurrence of dangerous events (hypo/hyperglycemia), etc. That being said, the main objectives of the self-management of diabetes is to improve the lifestyle and life quality of patients with diabetes; (2) Methods: We performed a systematic review based on articles that focus on the use of smart devices for the monitoring and better management of diabetes. The search was focused on keywords related to the topic, such as “Diabetes”, “Technology”, “Self-management”, “Artificial Intelligence”, etc. This was performed using databases, such as Scopus, Google Scholar, and PubMed; (3) Results: A total of 89 studies, published between 2011 and 2021, were included. The majority of the selected research aims to solve a diabetes management problem (e.g., blood glucose prediction, early detection of risk events, and the automatic adjustment of insulin doses, etc.). In these studies, wearable devices were used in combination with artificial intelligence (AI) techniques; (4) Conclusions: Wearable devices have attracted a great deal of scientific interest in the field of healthcare for people with chronic conditions, such as diabetes. They are capable of assisting in the management of diabetes, as well as preventing complications associated with this condition. Furthermore, the usage of these devices has improved illness management and quality of life.

## 1. Introduction

Diabetes is a persistent disease in which the level of sugar in the blood is high. It can be caused by either a lack or absence of insulin production, or by a loss of insulin effectiveness [1]. The principal hormone that regulates the uptake of glucose present in the blood by most cells (muscle and fat cells) is insulin. When insulin is not available in sufficient quantities, glucose can no longer be absorbed by the cells in the body that need it and therefore its normal use is disrupted [2].

This disease is one of the world’s fastest growing health problems of the twenty-first century, with the number of diabetics more than tripling in the last 20 years. According to the International Diabetes Federation (IDF), diabetes infected 463 million people globally in 2019, which means that 1 in 11 adults (20–79 years of age) has diabetes. Furthermore, The IDF estimates that 578 million adults will have diabetes by 2030, and 700 million by 2045 [3,4].

The main categories of diabetes are “type 1 diabetes (T1D)”, “type 2 diabetes (T2D)”, and “gestational diabetes (GDM)”. Type 1 diabetes affects around 8% of all patients of this disease. In this type, the body produces little or no insulin. The treatment of type 1 diabetes is insulin injection. T1D can affect people of any age, but it is mainly present in children and young adults. Type 2 diabetes is the most common and accounts for about 90% of all forms of diabetes, and it is most frequently diagnosed in elderly people. Generally, it is characterized by insulin resistance. Lastly, the type 3 gestational diabetes is a type of diabetes that first appears during pregnancy and usually disappears shortly after delivery [5,6,7].

Diabetes is a disease characterized by fluctuating blood sugar levels that increase or decrease. The persistence of this abnormality over a long period of time increases the likelihood that the patient with diabetes will develop other health problems. Most of these complications commonly occur in patients with type 1 or type 2 diabetes. We distinguish chronic diabetic complications into two broad categories: microvascular complications and macrovascular ones, the prevalence of which is much higher in the former than in the latter [8]. Among the microvascular complications are nephropathy, neuropathy, and retinopathy, whereas those of the macrovascular type are coronary heart disease (CHD), myocardial infarction, peripheral arterial disease (PAD), and stroke [9].

Over the last decade, there has been a tremendous increase in consumer contact with technology and artificial intelligence [10]. Consumers’ usage of wearable technology is part of the current technological revolution. Wearable devices, or simply wearables, are defined by Wright et al. [11] as smart computers integrated into various accessories, such as clothing, fashion accessories, smartwatches, and other everyday objects worn by customers. The application of these technologies in healthcare is rapidly increasing. This is due to the variety of sensors that these gadgets are equipped with, including those that detect sound, images, body movements, and ambient light levels [12].

A process called predictive analytics uses a range of machine learning algorithms, data mining techniques, and statistical approaches to find patterns or signals and predict future occurrences from current and past data. Predictive analytics, when applied to health data, can help make critical choices and predictions. Machine learning and regression techniques are used to perform this predictive analysis. This method aims to diagnose a disease as accurately as possible, to better care for patients, to make the best use of resources, and to achieve better clinical outcomes [13]. Machine learning is viewed as one of the most important aspects of artificial intelligence, as it allows for the design of computer systems that can learn from previous experience without the need for programming for every situation.

The combination of artificial intelligence approaches and advanced technologies, such as medical devices, wearable devices, and sensor technologies, could enable the development and implementation of better chronic disease management services [14]. In this paper, diabetes is the health problem addressed.

In recent years, new technologies have been created for the management of diabetes and its complications. In this article, in the form of a systematic literature review, we will give an overview and clearer idea of current technologies (smart wearables) as well as AI techniques used to help manage this disease. AI has substantial applications in four primary fields of diabetes care: automated retinopathy detection, clinical decision support, predictive population risk stratification, and patients’ self-tools [15,16].

The accumulated literature about diabetes management with different tools based on artificial intelligence is vast and difficult to grasp. In this article, we aim to classify and review the most relevant works. Our objective is to present in a clear and more relevant way the advancement of science in the field of diabetes management using smart devices and machine learning.

Previous reviews dealing with the topic of diabetes management (see Table 1) have mostly been focused on the use of just mobile applications. We even find, as mentioned in Table 1, that these reviews cover the subject in less depth. Other studies only focus on a specific type of diabetes or on a specific age group. The fact that we focused on reviewing each of the included studies individually, as well as providing detailed results for each, allows us to provide a more in-depth view, to the point of questioning aspects traditionally taken for granted.

This paper according to the sections below: methods describing the eligibility criteria for article selection, choice of data sources, and the study selection/data extraction process; the results section details the research topic, participants, metrics, smart device types and models, and AI approaches employed for each included study; and finally the conclusion offers prospective future pathways for smartwatch research.

## 2. Materials and Methods

A systematic review is an explicit and reproducible research methodology that identifies all associated experiments and also summarizes the state of the art, in order to answer one or more fundamental research questions on a particular topic [23,24].

In this section, we will present the detailed methodology utilized to conduct the systematic review, which was based on the guidelines described in the PRISMA method: preferred reporting items for systematic reviews and meta-analysis [25].

### 2.1. Eligibility Criteria

The eligibility criteria for the selection of articles listed below are also summarized in Table 2:

- Only papers written in English;
- Only papers published in the last 10 years (between January 2011 and May 2021) due to the fast technological developments in diabetes self-management;
- Only papers with diabetes management and its complications as the main topic;
- Only papers dealing with the management of type 1, type 2, or gestational diabetes;
- Only papers that focus on diabetes self-management using devices which are either portable or mounted on the body were included;
- Only papers addressing the topic with artificial intelligence (AI)-based techniques.

**Figure 1:**
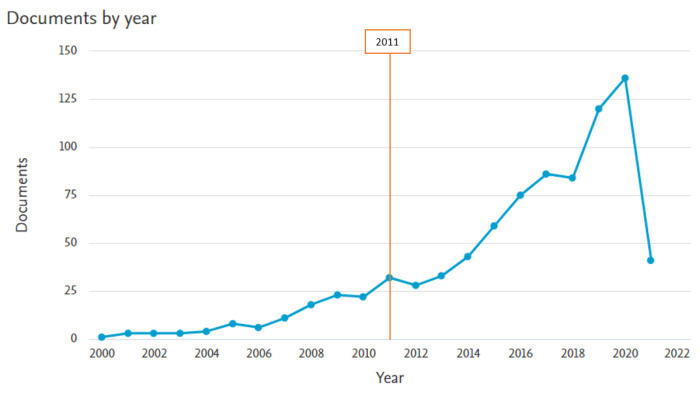
Distribution of documents by year (SCOPUS database).

Scientific interest in the use of wearable technologies in diabetes healthcare has increased over the last decade. The articles included in this review were published between 2011 and 2021. During this period (in the SCOPUS and PubMed databases), 716 articles were published, while the period before 2011 there were only 159 publications (Figure 1).

Papers were excluded if they: (i) were a short conference/congress abstract; (ii) were a review article; (iii) were published before 2011; or (iv) were not available in full text. In addition, studies that do not meet the criteria listed above are excluded from the review.

### 2.2. Data Sources and Search Strategy

For the identification and collection of articles related to the domains of interest of our systematic review, a literature search was performed in the Scopus and PubMed databases. We selected these electronic databases by reason of their relevance to the research subject and scope. The title, abstract, and keywords were the fields considered in the search queries. The searches were formulated using several keywords falling within the topics already described in the eligibility criteria, and by using Boolean operators (AND, OR, and NOT) in order to interrogate the electronic databases of scientific publications. As an example, the search strings implemented in Scopus and PubMed are provided in Table 3. In addition, more articles were obtained from the bibliographies of the identified papers.

The electronic search terms used were: (i) Device Type: “wearabl*”; “device*”; “smart devic*”; “watch”; “smartwatch”; “smart*”; “Portable”; "mobile”. (ii) Control Technologies: “intellig*”; “artificial”; “machine learning”; “AI”; “learn*”; “classification”; “regression”; “ANN”; “artificial neur*”; “net*”. (iii) Medical Domain: “diabet*”; “hypoglycem*”; “hyperglycem*”.

### 2.3. Study Selection

After querying the databases, we used EndNote^®^ X9.3.3 reference management tool to log references, to eliminate multiple records, and to create a unique database of references.

To select articles from the initial database, we applied a three-step process, as recommended by Stefana et al. [26] in “Inclusion and Exclusion Criteria” section:

1. Evaluation of the title;
2. Evaluation of the abstract and keywords;
3. Evaluation of the full text.

The aim was to remove irrelevant searches during stages (1) and (2), and then review the remaining documents using the above eligibility criteria during stage (3). Finally, in the eligibility phase, we assembled the included studies into our final database, and reported the main reason for the exclusion of other articles on the basis of given criteria.

**Figure 2:**
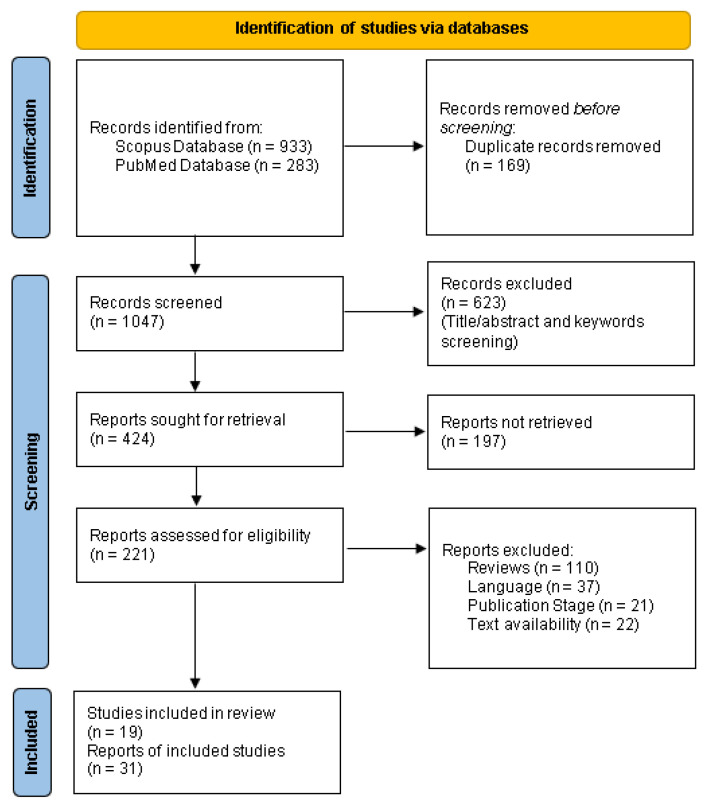
The PRISMA flow diagram.

The entire flowchart for the selection process, including identification, screening, eligibility, and inclusion, is shown in Figure 2 [27].

### 2.4. Data Extraction

For each article included, the following information was also included: (i) study focus, (ii) type of device, (iii) device model, (iv) participants information, (v) AI technologies used, (vi) approach used, (vii) parameters extracted, and (viii) technological challenges associated with the use of smart devices for diabetes management. The results were discussed in order to show the most recent status of wearable-device research for medical purposes (diabetes management).

## 3. Results

The selected research is 19 in number and was published between 2011 and 2021. The selection procedure started initially with 1216 articles and multiple elimination steps were performed. Firstly, 169 were eliminated, mostly due to being duplicates. Secondly, title/abstract screening reduced the remaining 1047 articles into 424 (excluding 623 records). From this number, 197 were excluded as we were not able to get the full text. Finally, 190 were removed for unfitting the inclusion criteria to bring about an outcome of 19 articles. The majority of the selected studies are aimed at solving one of the diabetes management problems: blood glucose prediction, early detection of risk events, automatic adjustment of insulin doses, etc. Wearable gadgets combined with artificial intelligence approaches have been used in this research. This systematic review concluded in 19 articles.

### 3.1. Diabetes Technology

Diabetes technology describes the hardware, devices, and software used by people with diabetes in managing the levels of blood glucose, avoiding the complications of diabetes, reducing the stress of living with diabetes, and making their lives better [28].

**Figure 3:**
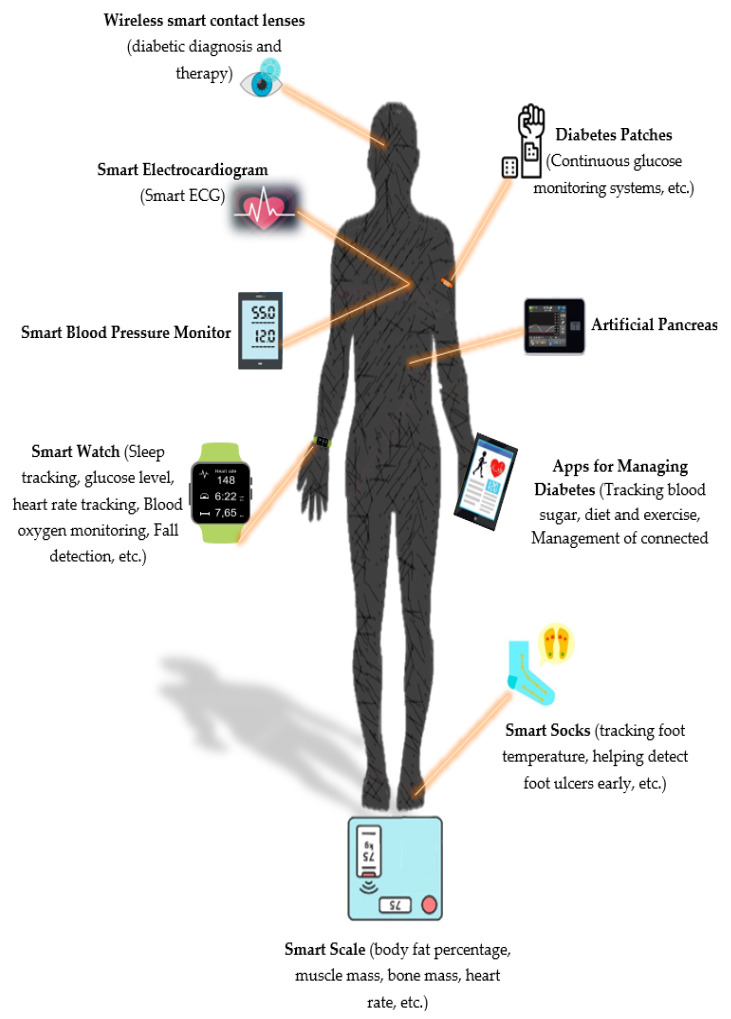
Wearable devices used to support patients with diabetes.(ECG: electrocardiography).

“Smart” glucose and blood pressure monitors, activity trackers, and scales have become the most widely used connected devices in the diabetes world. From “smart” socks, which are supposed to monitor foot temperature to prevent inflammation and ulcers, to wearable mini electrocardiographs (ECGs) connected to track cardiovascular health, the growing number and variety of devices for people living with diabetes is expected to revolutionize how the disease is managed (Figure 3) [29].

The use of smart technologies to manage diabetes remains a promising area of research that can significantly improve the quality of life for people affected by this disease [30].

### 3.2. Artificial Intelligence Techniques

Artificial intelligence (AI) is a field of computer science that focuses on developing technologies to do what normally needs to be performed by human intelligence. These programs can imitate or replicate cognitive behaviors or skills related to human intelligence, such as reasoning, problem solving, and learning. Today, it is one of the most rapidly developing branches of computing and computation, with an important potential impact on healthcare [31].

In the field of health, one of the most significant branches of AI is machine learning. Machine learning is a field of artificial intelligence (AI) that concentrates on developing algorithms capable of learning from data and improving their performances over time without being programmed to do so [32].

**Figure 4:**
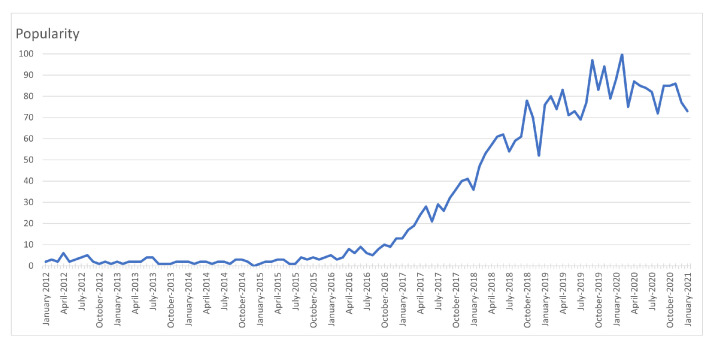
The worldwide popularity rating for the term “ai healthcare” within a range of 0 (min) to 100 (max) in time, where the x-axis represents the timestamp information and the y-axis shows the corresponding score [33].

The use of AI in healthcare is becoming increasingly more popular, as shown in Figure 4, based on Google Trends data over the past decade [33]. Here, the x-axis indicates specific dates, and the y-axis represents the corresponding popularity ranking, which ranges from 0 (minimum) to 100 (maximum). Based on Figure 4, it can be seen that the popularity indicator values for the use of the term “ai healthcare” carried low values in 2012, but has been gradually increasing since.

#### 3.2.1. Types of Machine Learning Techniques

**Figure 5:**
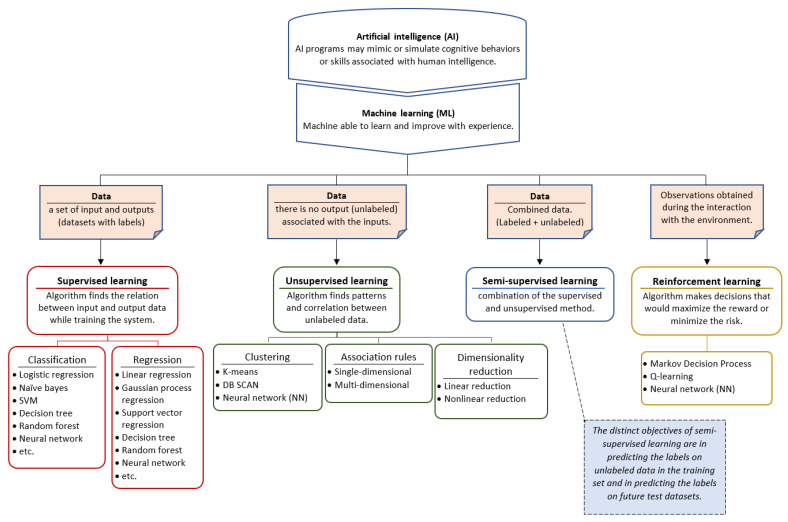
Machine learning models and algorithms.

Machine learning (ML) algorithms are mainly divided into four categories [34] (Figure 5). These are:

1. Supervised Learning: where the system deduces a function from labeled training data.
2. Unsupervised Learning: where the training system attempts to deduce the structure of unlabeled data.
3. Semi-Supervised Learning: can be described as a combination of the supervised and unsupervised methods mentioned above, as it works on both labeled and unlabeled data.
4. Reinforcement Learning: where the system interacts with a dynamic environment [32,35].

Figure 5 shows the taxonomy of ML techniques [36].

**Figure 6:**
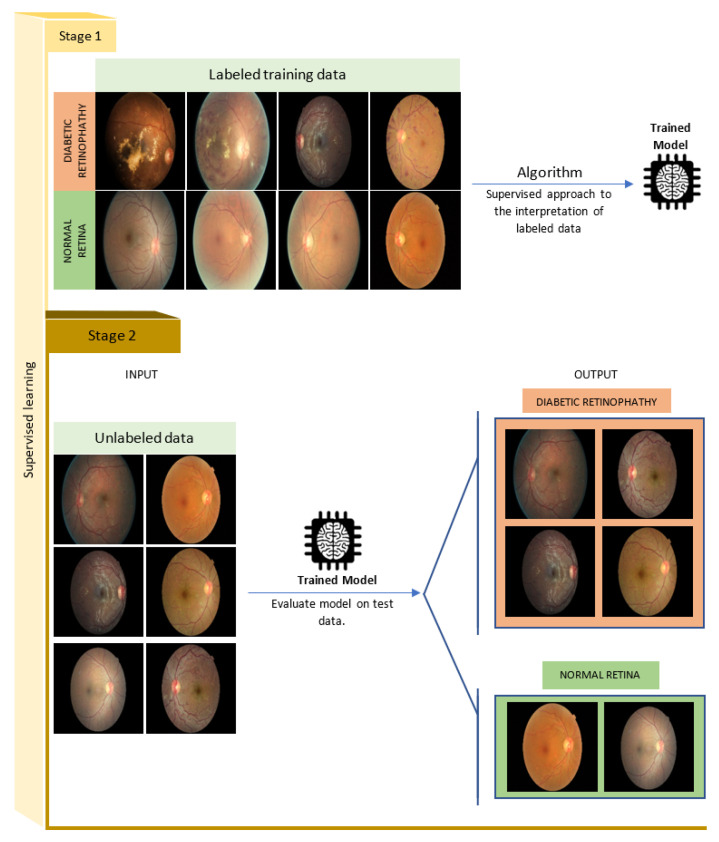
Supervised learning model. The supervised method consists of learning from labeled data, where each input data is associated with its output label (Stage 1). Then, the algorithm is validated on another set of unlabeled data, which the machine has not seen before (Stage 2) (images sourced from Kaggle’s platform).

**Supervised Learning:** One of the most powerful data analysis approaches in machine learning is the supervised learning model. In this type of learning, the system tries to learn from the labeled data the corresponding function (*f*) that maps an input (*x*) to an output (*y*) (Figure 6) [37]. At the end of the learning process, we will get a function:
  f:x→y
  which the algorithm will use for making predictions on unlabeled data [34]. The output in this type of learning is obtained by data classification or regression (value prediction). Classification algorithms, such as the examples given in Figure 5, are designed to predict distinct classes, while regression algorithms predict numerical values (e.g., the prediction of blood glucose values) [38].

Figure 6 is an application of a supervised learning model, which allows a classification of diabetic retinopathy fundus images [39]. This example is based on images obtained on the Kaggle platform [40].

**Figure 7:**
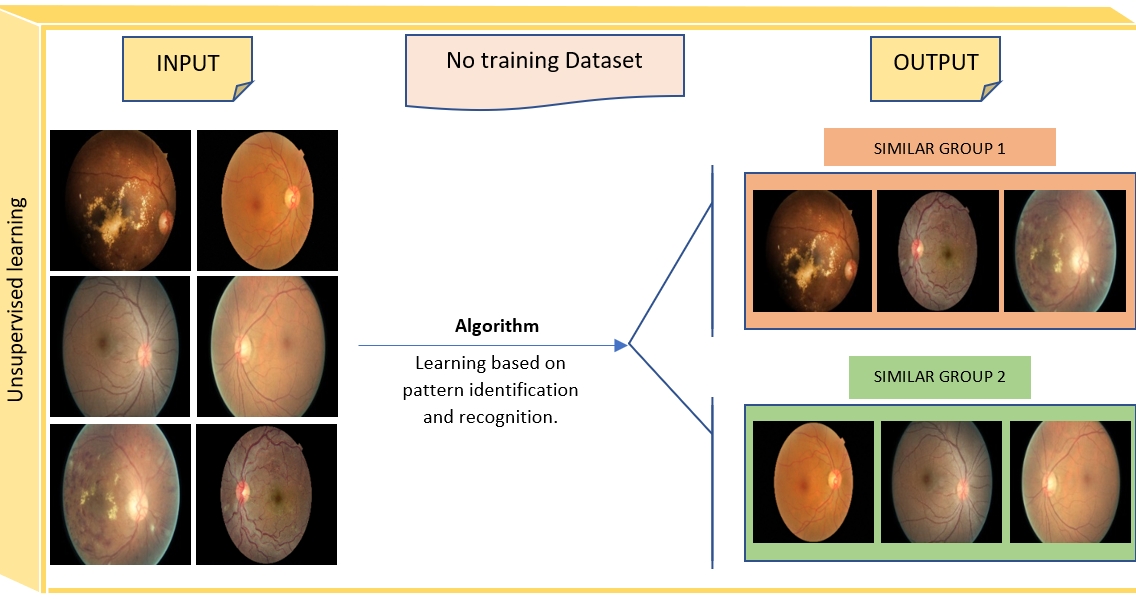
Unsupervised learning model (images sourced from Kaggle’s platform).

**Unsupervised Learning:** Unlike supervised machine learning, in unsupervised machine learning, the system attempts to find and discover the hidden data structure or the relationships between variables with no preexisting labels or specifications [41]. The training data for this method consists of a set of data that is not labeled, categorized, or classified (Figure 7) [42]. The output of this type of learning is obtained by using one of the following main ML methods: clustering, association rules, and dimensionality reduction (Figure 5). The difference between clustering and classification is that clustering attempts to group a set of objects and determine if there is a relationship between these objects (no pre-defined classes), while classification attempts to classify new simple objects into known classes [43]. Clustering can be used in healthcare, for example, to identify groups of cohesive and well-separated patients with diabetes who share similar profiles (e.g., age and gender) as well as common clinical histories [44].

**Semi-Supervised Learning:** Semi-supervised learning is a combination of supervised and unsupervised methods. The learning in this kind of algorithm uses labeled and unlabeled data (Figure 5) [45]. This method is generally used to solve problems where the number of available data is large and only a very limited set of labeled data is present [46].

One of the application areas of semi-supervised learning is in healthcare. To illustrate, Whu et al. developed a diabetic predictive model using semi-supervised learning (the Laplacian support vector machine (LapSVM)) [47].

**Figure 8:**
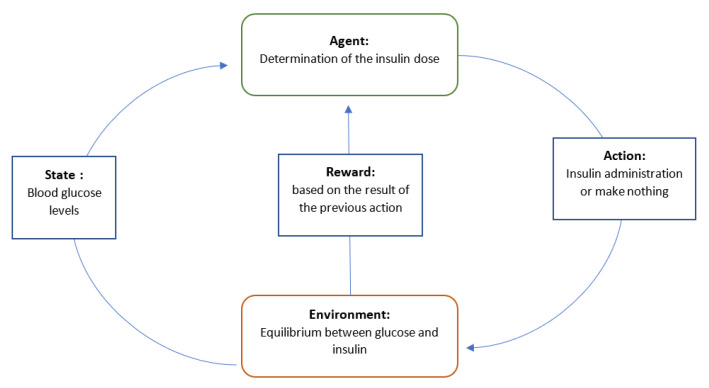
Reinforcement learning models for diabetes. The state changes cause the agent to act, resulting in a modification of the environment. A numerical reward is provided to the agent by the environment, influencing the agent’s next action with the next state.

**Reinforcement Learning:** The reinforcement learning method is a reward- or penalty-based method [48]. Indeed, its principal objective is to exploit the information and observations obtained from the interaction with the environment, in order to maximize the reward or minimize the risk [49]. The reinforcement learning algorithm (agent) is continually learning by interacting with the environment, aiming to explore the full range of possible states and to make the most proper decisions [34]. The agent’s actions affect the environment’s state (Figure 8).

The integration of reinforcement learning in the healthcare industry has often led to better outcomes [50]. As an example, this type of algorithm is used in people with diabetes to enhance their health and blood sugar control [51]. Yom-Tov et al. [52] developed a mobile application that aims to motivate people with diabetes to be physically active. This application was associated with a learning algorithm, which was able to predict better messages that would encourage patients to exercise.

#### 3.2.2. Different Techniques Used by ML

**Support Vector Machine:** A support vector machine (SVM) was developed in the 1990s. As a simple and important process, this method is used to perform machine learning (ML) tasks. A set of training samples is provided throughout this procedure, with each sample split into distinct categories. Support Vector Machine (SVM) is a type of machine learning algorithm that is commonly used to solve classification and regression issues [53].
**Bayes Classification:** Statistical classifiers are an example of Bayesian classifiers. Based on a given class label, naive Bayes determines the probability of class membership [54]. It conducts a single scan of data, making categorization simple.
**Decision Tree:** A decision tree (DT) is a classification method that consists of an internal node and a leaf node that has a class label. The decision tree’s (DT) top nodes are referred to as root nodes. This technique is popular because it is simple to construct and does not require any parameters [55].
**K-Nearest Neighbors:** The K-nearest neighbors method is a popular method for classifying data. We can calculate the distance measurement from N training samples using this approach [56].
**Logistic Regression (LR):** Logistic regression is a typical probabilistic-based statistical model used to address classification problems in machine learning. To estimate probabilities, logistic regression generally uses a logistic function. It is able to deal with high-dimensional datasets and performs well when the dataset can be split linearly. A key disadvantage of logistic regression is the assumption of linearity between the dependent and independent variables. It may be used to solve both classification and regression issues. However, it is most often employed to solve classification problems [57].
**Adaptive Boosting (AdaBoost):** AdaBoost is an ensemble learning technique that uses an iterative strategy to improve weak classifiers by learning from their failures. Adaboost employs “sequential ensembling” as opposed to the random forest, which employs “parallel ensembling”. It generates a strong classifier by assembling multiple low-performing classifiers to get a high-accuracy classifier. AdaBoost is best utilized to improve the performance of decision trees and the base estimator on binary classification tasks [58].

#### 3.2.3. Examples of Machine Learning in Everyday Life

The technique of machine learning (ML) is a universal concept applied in a large number of fields; however, we still ignore the fact that we use it regularly.

Recently, machine learning methods are being used to identify and successfully filter junk emails. Yahoo’s basic methods for finding spam messages include URL (uniform resource locator) filtering, email body text, and customer spam complaints. Contrary to Gmail, Yahoo filters email messages based on domains, not IP addresses (Internet Protocol addresses) [59].

Plagiarism detection is an excellent example of AI application in the academic domain. One of the approaches used to combat plagiarism is the multi-agent machine learning (MML) system [60].

Netflix is an outstanding example of a good ML program in terms of daily usage. Based on the habits of its users, for example, the platform recommends a series of titles and visual content tailored specifically to the person watching [61].

**Figure 9:**
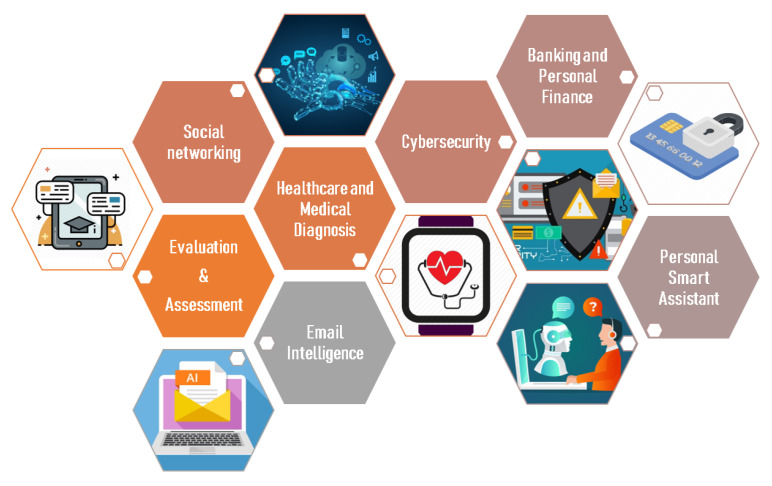
Examples of machine learning (ML) applications in everyday life.

Figure 9 illustrates some of the machine learning applications in our daily life.

### 3.3. Artificial Intelligence and Diabetes

The field of artificial intelligence (AI) is rapidly evolving and its application to diabetes has the potential to revolutionize the approach to diagnosing and treating this disease [62]. Furthermore, the integration of AI with smart devices, such as medical devices, wearables, smartphones, and sensor technology, will allow for the building of a machine capable of supervising and monitoring people with diabetes continuously [63].

Machine learning has the potential to revolutionize the way of managing diabetes, by permitting faster and more accurate decision making and improving existing healthcare standards. Table 4 summarizes the various AI application fields in the management of diabetes.

### 3.4. Search Results

Table 5 displays smart device types and models, the various sensor-based methods used, research subjects, participant information, and the AI technologies and strategies employed. Table 6 displays the study’s objectives and findings. Sixteen of the nineteen studies (84%) were about patients. One of the remaining three studies involved the creation of a smartphone application to assist people with type 1 diabetes in counting the carbohydrates in their diet, so the database included images of various meal components; the other two studies used virtual subjects to predict blood glucose levels as well as critical events.

The introduction of new technologies such as continuous glucose monitoring (CGM) devices, smart wearables (bracelets, smartwatches, smart clothing, and patches, etc.), and artificial pancreas (AP) development, and, arguably, the use of the data collected from these new tools, have revolutionized the overall diabetes management ecosystem over the past decade [94]. There are powerful AI methods for designing models that aim to prevent events such as hypoglycemia, predict the value of blood glucose levels, and predict the right amount of insulin to administer, all with the goal of improving the quality of life and illness management of people with diabetes, designing personalized management for each patient [95], and saving them from complications due to diabetes and early mortality [75].

Advanced tools that are used for diabetes management include continuous glucose monitors (CGM). Nine studies have used this device to collect the data needed for the development of smart systems [77,79,81,82,84,86,89,90,92]. Allam et al. [79] collected the inputs for a system that will be able to forecast future glucose concentration levels with prediction horizons (PH) of 15, 30, 45, and 60 min using a continuous glucose monitoring (CGM) device. Similarly, Pustozerov et al. [84] employed the CGM to develop a blood glucose prediction model to successfully support women with gestational diabetes (GDM).

New deep learning algorithms were developed to automate the diagnosis of DR. Retinal screening based on AI is an attainable, precise, and highly accepted method for the detection and monitoring of diabetic retinopathy. A sensitivity (DR: 83.3%, RDR: 93%) and a specificity (DR: 95.5%; DRR: 92.5%) were reported for both the automated screening of referable diabetic retinopathy and diabetic retinopathy, as shown in the study conducted by Sosale et al. [93]. Convolutional neural networks (CNNs) and a smartphone fundus camera were used to develop such a system.

Artificial intelligence enables patients with diabetes to make daily decisions about diet and activity. There are many applications designed to analyze the contents of meals and provide detailed information on the nutritional and caloric value of foods. Anthimopoulos et al. [78] developed an application for patients to help them assess the quality and caloric value of the food they eat. The management of diabetes is more effective when patients take a picture of their own food and evaluate what they eat.

Physical exercise has been identified as one of the most effective initial prevention strategies for diabetes in high-risk individuals. With wearable devices that record the number of steps and the duration and intensity of activities, daily activity levels can be tracked. These technologies allow for the monitoring of daily activity and may encourage a person to include activity as part of their routine to better stabilize their blood glucose levels. Yom-Tov et al. [52] designed a reinforcement learning algorithm-based system that aims to personalize messages for each patient’s situation in order to better encourage them to practice sports activities.

Among all the articles included, different techniques were employed with the aim of establishing a device that could properly manage or assist in the management of diabetes. By analyzing the results of the different papers, we can find that the most commonly employed function of the smart devices is the “prediction of blood glucose levels”, with a percentage of (36.84%). We can also see that the most used approach is classification, with a percentage of 52.6% compared to regression, which was used in 47.4% of the studies. However, it is necessary to note that in future clinical applications it will be required to perform longitudinal studies in order to measure both the inter- and intra-subject variability. On the other hand, to conduct such studies can be challenging because the large-scale deployment of wearables can be financially and technically challenging.

## 4. Conclusions

Wearable technologies have sparked a lot of scientific interest in the field of healthcare, especially for patients with chronic conditions, such as diabetes, during the last two decades. They are capable of assisting in the treatment of diabetes, as well as preventing problems connected to the condition. Furthermore, the usage of these devices has improved diabetes control as well as quality of life.

This article provides a systematic review of intelligent systems developed for use as diabetes-control instruments. A thorough screening process identified 19 articles. These papers were evaluated and studied to obtain the various information needed to answer the review questions. These included the types and models of the smart devices used, different sensor-based methods, participant information, and the AI technologies and approach used.

In summary, it can be said that new digital technologies, big data-based analytics, and the application of AI to diabetes data will revolutionize the way diabetes and diabetes-related complications are treated, as well as their prevention and control.

## Figures and Tables

**Table 1:** Previous reviews dealing with the topic of diabetes management (Published on 2021).

Title	Year	Limitations
Implementation and impact of mobile health (mHealth) in the management of diabetes mellitus in Africa: a systematic review protocol [17]	2021	- Related to mHealth and targets a specific region and type of diabetes. It also did not provide a detailed analysis of each included article.
Effectiveness of mobile applications in diabetic patients’ healthy lifestyles: a review of systematic reviews [18]	2021	- Presents only the management of diabetes using mobile applications
Mobile and wearable technology for the monitoring of diabetes-related parameters: Systematic review [19]	2021	- Focused on the devices rather than machine learning
Mobile apps for the treatment of diabetes patients: a systematic review [20]	2021	- Presents only the management of diabetes using mobile applications.
Effects of offloading devices on static and dynamic balance in patients with diabetic peripheral neuropathy: a systematic review [21]	2021	- Deals with only part of the fields of diabetes management.
Mobile app interventions to improve medication adherence among type 2 diabetes mellitus patients: a systematic review of clinical trials [22]	2021	- Presents only the management of diabetes using mobile applications. - Focused only on one type of diabetes.

**Table 2:** Summary of inclusion criteria.

Criteria	Definition
Language of papers	English
Years considered	Between January 2011 and May 2021.
Subject	The use of smart devices in the management of diabetes.
	• Computer science.
Fields	• Medicine.
	• Artificial intelligence (AI).
	• Type 1 diabetes (T1D).
Type of diabetes considered	• Type 2 diabetes (T2D).
	• Gestational diabetes (GDM).
Age of participants	No restrictions related to age.
Types of devices	• Portable. • Mounted on the body.

**Table 3:** Search strategies for the selected databases.

Database	Search Query
SCOPUS	TITLE ((“wearabl*” OR “device*” OR “smart devic*” OR “watch” OR “smartwatch” OR “smart” OR “Portable” OR “mobile”) AND (“diabet*” OR “hypoglycem*” OR “hyperglycem*”) AND NOT (“systematic review”)) AND ALL((“wearabl*” OR “device*” OR “smart devic*” OR “watch” OR “smart watch” OR “Portable” OR “mobile”) AND (“diabet*” OR “hypoglycem*” OR “hyperglycem*”) AND (“intellig*” OR “artificial” OR “machine learning” OR “AI” OR “learn*” OR “classification” OR “regression” OR “ANN” OR “artificial neur*” OR “net*”)) AND (LIMIT-TO (PUBSTAGE,“final” )) AND (LIMIT-TO (LANGUAGE,“English” )) AND (EXCLUDE (DOCTYPE,“re” )) AND (LIMIT-TO (PUBYEAR,2021) OR LIMIT-TO (PUBYEAR,2020) OR LIMIT-TO (PUBYEAR,2019) OR LIMIT-TO (PUBYEAR,2018) OR LIMIT-TO (PUBYEAR,2017) OR LIMIT-TO (PUBYEAR,2016) OR LIMIT-TO (PUBYEAR,2015) OR LIMIT-TO (PUBYEAR,2014) OR LIMIT-TO (PUBYEAR,2013) OR LIMIT-TO (PUBYEAR,2012) OR LIMIT-TO (PUBYEAR,2011))
PubMed	(((((((“wearabl*”[Title] OR “device*”[Title] OR “smart devic*”[Title] OR “watch”[Title] OR “smartwatch”[Title] OR “smart*” OR “Portable”[Title] OR “mobile”[Title])) AND ((“diabet*”[Title] OR “hypoglycem*”[Title] OR “hyperglycem*”[Title] ))) AND ((“wearabl*” OR “device*” OR “smart devic*” OR “watch” OR “smart watch” OR “Portable” OR “mobile” ))) AND ((“diabet*” OR “hypogly-cem*” OR “hyperglycem*” ))) AND ((“intellig*” OR “artificial” OR “machine learning” OR “AI” OR “learn*” OR “classification” OR “regression” OR “ANN” OR “artificial neur*” OR “net*” ))) NOT (“systematic review”[Title])) AND ((“2011”[Date—Publication]: “2021/04/18”[Date—Publication])) AND (English[Language])

**Table 4:** AI applications and ML techniques used in various domains of the management of diabetes.

Domain of use	Applications	Type of ML Methods	ML Technique Used	Year	Reference
BG Prediction	Predict blood glucose values to provide early warnings.	Regression	ANN	2012	[64]
			SVM, RA, ANN	2013	[65]
			SVR	2013	[66]
			KNN, RF	2017	[67]
	Early detection and diagnosis of diabetes.	Classification	SVM	2013	[68]
Detection of Adverse Glycemic Events (Hypo/Hyper)	Early detection and rapid response to risky glycemic events.	Classification	ANN	2013	[69]
			SVM	2013	[70]
			RF	2014	[71]
			ANN	2016	[72]
Advisory Systems	Identifying clusters of people with similar forefoot loading patterns.	Clustering	K-means	2013	[73]
	Identification of renal risk clusters in African American women with type 2 diabetes and categorize the risk groups (low risk and high risk).	Clustering	K-means	2015	[74]
	Prediction of the risk for future occurrence of microvascular complications (nephropathy, neuropathy, and retinopathy).	Classification	RF, LR	2018	[75]
Detection of Exercise	Automatic detection of the type (aerobic and anaerobic exercise) and duration of the exercises performed.	Classification	KNN	2015	[76]
	The automation of exercise detection and the management of insulin and glucagon dosages during activity.	Regression	Linear Regression	2015	[77]
Lifestyle and Daily- Life Support in Diabetes Management	Help patients with type 1 diabetes to count carbohydrates in food using the smartphone (automatic detection).	Clustering	Hkmeans	2015	[78]
		Classification	SVM		

**Table 5:** The description of selected articles based on smart device types, models, study focus, participants, AI technologies used, and approach used.

Title	Doi	Year	Authors	Study Focus	Types of Devices	Devices Model	Sensors	Participants	AI Technologies Used	Approach Used
A Recurrent Neural Network Approach for Predicting Glucose Concentration in Type-1 diabetic patient	10.1007/978-3-642-23957-1_29	2011	Allam et al. [79]	Blood glucose prediction	Continuous Glucose Monitoring (CGM) System	Gaurdian® Real Time CGM system (MedtronicMinimed)	CGM sensor (Glucose sensor)	n = 9, type-1 patient with diabetes (T1D)	Recurrent neural network (RNN)	Regression
Electrocardiographic Signals and Swarm-Based Support Vector Machine for Hypoglycemia Detection	10.1007/s10439-011-0446-7	2012	Nuryani et al. [80]	Hypoglycemia detection using the ECG parameters as inputs	The Siesta System	COMPUMEDICS	Not specified	n = 5, patient with diabetes with age of 16 ± 0.7 years	Support vector machine (SVM)	Classification
Blood Glucose Level Prediction using Physiological Models and Support Vector Regression	10.1109/ICMLA.2013.30	2013	Bunescu et al. [81]	Blood glucose prediction	Continuous Glucose Monitoring (CGM) System	Not specified	CGM sensor (Glucose sensor)	n = 10, T1D patients	Support vector regression (SVR)	Regression
					Smartphone					
Jump neural network for online short-time prediction of blood glucose from continuous monitoring sensors and meal information.	10.1016/j.cmpb.2013.09.016	2014	Zecchin et al. [82]	Blood glucose prediction	Continuous Glucose Monitoring (CGM) System	DEXCOM SEVEN PLUS	CGM sensor (Glucose sensor)	n = 20, T1D patients	Jump neural network	Regression
Incorporating an Exercise Detection, Grading, and Hormone Dosing Algorithm into the Artificial Pancreas Using Accelerometry and Heart Rate	10.1177/ 19322968 15609371	2015	Jacobs et al. [77]	Detection of exercise activity Automatic adjustment of insulin/ Glucagon doses	- CGM system	- Dexcom G4	- CGM Sensors	n = 13, T1D patients	Linear Regression	Regression (estimate EE in kilocalories/ minute)
					- Android smartphone	- Google Nexus	- 3-Axis Accelerometer			
					- Biopatch	- Zephyr Biopatch (Zephyr Technology)	- Heart Rate Sensors			
					- Insulin pump	- Not specified				
Computer Vision-Based Carbohydrate Estimation for type 1 Patients with Diabetes Using Smartphones	10.1177/ 19322968 15580159	2015	Anthimopoulos et al. [78]	Measurement of the caloric intake of food	Smartphone (application)	Not specified	Accelerometer	-	Hierarchical k-means	Clustering
							Gravity sensor			
							Camera		SVM	Classification
Classification of Physical Activity: Information to Artificial Pancreas Control Systems in Real Time	10.1177/ 19322968 15609369	2015	Turksoy et al. [76]	Automatic identification of the type and intensity of exercise	Chest Band	Bioharness-3 (Zephyr Technology, Annapolis MD)	Heart Rate Sensors	n = 8, subjects are tested (5 with T1D, 3 without T1D)	SVM	Classification
					Fitmate Pro	COSMED	Breathing sensor			
Non-Invasive Blood Glucose Detection System Based on Conservation of Energy Method	10.1088/1361-6579/aa50cf	2017	Zhang et al. [83]	Blood Glucose Prediction	Non-Invasive BG Detection System	Not specified	-Temperature Sensor. -Radiation Thermometer. -Humidity Sensor. -Photoelectric Detector (PD). -Dual Wavelength LEDs.	n = 180, 45 patient with diabetes, 91 senior citizens (36 patients with hypertension), 54 adults in good health	Decision Tree Back propagation neural network	Classification Regression
Encouraging Physical Activity in Patients with Diabetes: Intervention Using a Reinforcement Learning System	10.2196/jmir.7994	2017	Yom-Tov et al. [52]	Improving health and blood sugar control.	Smartphone	Android Smartphone	Accelerometer	n = 27 sedentary type 2 diabetes patients	Linear Regression	Regression
				Motivate people with diabetes to engage in sports activities.						
Development and Evaluation of a Mobile Personalized Blood Glucose Prediction System for Patients with Gestational Diabetes Mellitus	10.2196/mhealth.9236	2018	Pustozerov et al. [84]	-Blood Glucose Prediction, -Assistance to Gestational Diabetes Mellitus (GDM) patients,	-Mobile App, -Continuous Glucose Monitoring (CGM) System,	Medtronic iPro	Enlite sensors (Medtronic, Minneapolis, MN, USA)	n = 62 participants (48 pregnant women with GDM and 14 women with normal glucose tolerance)	Linear Regression	Regression
5G-Smart Diabetes: Toward Personalized Diabetes Diagnosis with Healthcare Big Data Clouds	10.1109 /MCOM.2018 .1700788	2018	Chen et al. [85]	Early detection and prevention of diabetes	Blood glucose device	Not specified	Not specified	n = 9594, 469 diabetes patients and 9081 normal persons	(Ensemble learning) Combination of: - Decision Tree, - ANN and - SVM.	Classification
					Smartphone					
					Wearable 2.0 (i.e., smart clothing)					
					Intelligent app					
Classification of Postprandial Glycemic Status with Application Insulin Dosing in Type 1 Diabetes—An In Silico Proof of Concept	10.3390/bs19143168	2019	Cappon et al. [86]	-Predict the future glycemic status in the postprandial period.	Continuous Glucose Monitoring (CGM) System	Not specified	Glucose sensor	Data of 100 virtual adult subjects	XGB-Extreme Gradient Boosted Tree Model.	Classification (hyperglycemia, euglycemia, or hypoglycemia)
				-Adjusting the insulin bolus according to the predicted glycemic status.						
Diabetes Care in Motion: Blood Glucose Estimation Using Wearable Devices	10.1109/MCE.2019.2941461	2019	Tsai et al. [87]	Prediction of blood glucose levels using the PPG signal	Wearable Health Device (Wristband)	Glutrac	Optical Sensors	n = 9 participants with type 2 diabetes, (3 Males, 6 Females)	Random forest Adaboost Regression	Regression
Classification of Fatigue Phases in Healthy and Diabetic Adults Using Wearable Sensor	10.3390/s20236 897	2020	Aljihmani et al. [88]	* Recognizing Rest/Effort Tasks. * Detection of early and late fatigue states.	- 3-axial accelerometer -Arduino	-ADXL 355 -UNO R3(Adafruit)	Accelerometer	n = 40 right-handed adults (19 males and 21 females), (20 healthy, 20 subjects with T1DM)	Ensemble Classifier Based on Random Subspace K-NN	Classification
Towards Wearable-based Hypoglycemia Detection and Warning in Diabetes	10.1145 /3334480 .3382808	2020	Maritsch et al. [89]	Hypoglycemia detection	Smartwatch	Empatica E4	Optical Sensor	n = 1 one otherwise healthy individual with T1DM	Gradient Boosting Decision Tree (GBDT)	Classification
							Three-Axis Accelerometer			
							CGM sensor			
Feature-Based Machine Learning Model for Real-Time Hypoglycemia Prediction	10.1177/1932296 820922 622	2020	Dave et al. [90]	Prediction of hypoglycemic events.	-CGM System -Insulin Pumps	-DEXCOM G6 -T-SLIM: X2	CGM sensor (Glucose sensor)	n = 112 patients	Random Forests	Classification
Potential Predictors of Type-2 Diabetes Risk: Machine Learning, Synthetic Data and Wearable Health Devices	10.1186 /s12859-020-03763-4	2020	Stolfi et al. [91]	Estimation of the risk of progression from a healthy state to a pathological state.	-Smart Phones, -Tablets, -Wearable Devices, and -Smartwatches	Not Specified	Not Specified	n = 46,170 virtual subjects	Random Forest	Regression
A Smart Glucose Monitoring System for Patient with Diabetes	10.3390/electronics 9040678	2020	Rghioui et al. [92]	-Diabetic Disease Monitoring, -Diabetic Assistance, -Predictions of Blood Glucose Levels	-Arduino Nano board -Smartphone -Smartwatches -Continuous Glucose Monitoring (CGM) System	Not Specified	-Glucose Sensor, -Motion Sensor, -Temperature Sensor, -Bluetooth.	n = 55 diabetic patients (39 men and 16 women)	Naive Bayes (NB), J48 Algorithm, Random Tree, ZeroR, SMO(sequential minimal optimization), and OneR algorithms	Classification
Simple, Mobile-Based Artificial Intelligence Algorithm in the Detection of Diabetic Retinopathy (SMART) study	10.1136/bmjdrc-2019-000892	2020	Sosale et al. [93]	Diagnosis of diabetic retinopathy (DR)	-Smartphone, -Fundus On Phone camera	-IPhone6, -Remidio Innovative Solutions	Camera	n = 900 individuals (252 had DR)	Convolutional Neural Networks (CNN).	Classification (DR present or absent)

NOTE: Diabetic retinopathy (DR), Referable Diabetic Retinopathy (RDR), Gestational Diabetes Mellitus (GDM), Type-1 patient with diabetes (T1D), Type-2 patient with diabetes (T2D). Continuous Glucose Monitoring (CGM), Naive Bayes (NB), Convolutional Neural Networks (CNN), Random Forest (RF), Logistic Regression (LR), Gradient Boosting Decision Tree (GBDT), Recurrent neural network (RNN), Support Vector Machine (SVM), Support Vector Regression (SVR), Artificial Neural Networks (ANN).

**Table 6:** Summary of each selected article.

Authors	Summary of Study Results
Allam et al. [79]	In this paper, a new approach for predicting future glucose concentration levels with prediction horizons (PH) of 15, 30, 45, and 60 min is proposed, using a recurrent neural network (RNN) and data collected from a continuous glucose monitoring (CGM) device. These predicted glucose levels can be used to set early hypoglycemia/hyperglycemia alerts to define adequate insulin doses. The suggested technique’s outcomes are assessed and compared to those produced from a feed-forward neural network prediction model (NNM). For relatively large prediction horizons, the results show that the RNN outperforms the NNM in predictions.
Nuryani et al. [80]	In this paper, a hybrid swarm-based support vector machine (SVM) method for hypoglycemia diagnosis is created by utilizing ECG values as inputs. A particle swarm optimization (PSO) approach is suggested in this method to optimize the SVM to identify hypoglycemia. With a sensitivity of 70.68 %, our novel SVM-RBF swarm-based hypoglycemia detection method outperforms the competition.
Bunescu et al. [81]	A machine learning model was designed to alert people with diabetes to impending changes in their blood sugar levels 30 min and 60 min in advance, giving them enough time to take preventive measures. For this purpose, a support vector regression (SVR) model was employed. This approach takes as input previous blood glucose readings obtained with a continuous glucose monitoring (CGM) device, as well as daily events such as insulin boluses and meals.
Zecchin et al. [82]	Development of an intelligent system able to accurately predict the future blood glucose level of diabetic patients with a time horizon of 30 min. This technique is based on a feed-forward NN, whose inputs are linked directly to the first hidden layer and the output neuron. This approach takes as input the CGM data and the amount of carbohydrates that the patient provides with their meal. The results obtained confirmed that this method provides a highly reliable prediction of glucose concentration.
Jacobs et al. [77]	The author demonstrates (1) the efficacy of an accelerometer and heart rate sensor for automated exercise detection, and (2) proposes a new algorithm for automated adjustment of insulin and glucagon dosages in response to exercise in this paper. This was based on a validated linear regression model that took the accelerometer and heart rate as inputs and provided energy expenditure (EE) as an output. With this model, the detection of the exercise event was possible with a sensitivity of 97.2% and a specificity of 99.5%.
Anthimopoulos et al. [78]	Development of a smartphone application to assist people with type 1 diabetes in counting carbs in diet. The identification of the different elements of the plate, the calculation of the proportions of the different parts and the estimation of the caloric intake of the meals are all actions performed using the images taken by the smartphone, the previous results, and the data provided by the USDA nutritional database. The assessment of the proposed system resulted in an average absolute percentage error in carbohydrate estimation of 10 ± 12%.
Turksoy et al. [76]	Development of a classification system able to detect automatically, in real time, both the type and intensity of exercise, and to classify it as aerobic or anaerobic. This system relied on the KNN algorithm, which took data from the Bioharness-3 chest belt as input. The sensitivity was 98.7 % on average. The use of biometric data and real-time classification of the intensity and type of exercise can provide helpful information to an AP for the prevention of hypoglycemia and hyperglycemia caused by exercise.
Zhang et al. [83]	Development of a non-invasive blood glucose detection device with high accuracy, low cost, and continuous glucose monitoring. This technique combines the energy conservation method with a sensor integration module that collects physiological data including blood oxygen saturation (SPO2), blood flow velocity, and heart rate. The model’s technique uses a decision tree and a back propagation neural network to classify glucose levels into three categories and train distinct neural network models for each. The system’s accuracy is 94.4%.
Yom-Tov et al. [52]	Research study to help patients with type 2 diabetes increase their physical activity. To this end, patients are given personalized messages based on each individual using reinforcement learning algorithms. In this paper, a linear regression algorithm with interactions was used to predict the change in activity from day t to the day t + 1, in order to select the appropriate feedback message to send.
Pustozerov et al. [84]	Development and implementation of a mobile technology-based system for data analysis, blood glucose prediction, and assistance to gestational diabetes mellitus patients (GDM) through a mobile application. The personalized recommendations are based on the results of blood glucose predictions. This mobile application was created using the Java programming language. On the other hand, blood glucose prediction was obtained using a linear regression model. This kind of model was chosen due to its high interpretability, simplicity, quick tweaking, and appropriate accuracy. Overall, 62 women participated in the study, including 48 pregnant women with GDM, and 14 others without diabetes.
Chen et al. [85]	Development of an intelligent system called 5G-Smart Diabetes, capable of predicting blood glucose levels, providing a personalized diagnosis, and suggesting a suitable treatment for the patient. An intelligent application has also been developed to communicate with all kinds of sensing devices, in order to provide patients with better services. In this study, three classical ML algorithms—decision tree, SVM, and artificial neural networks (ANN)—were used, to create alternative models for diabetes diagnosis. By combining the three algorithms, better prediction performance is obtained for the combined model than for each individual model.
Cappon et al. [86]	Development of a novel intelligent approach to classify postprandial glycemic status during meals (i.e., hypoglycemia, hyperglycemia, and euglycemia), and use its prediction to adapt the delivery of the mealtime insulin bolus. This method is based on the use of a classification technique, namely the XGB (extreme gradient boosted tree) model, able to predict the future glycemic state in the postprandial period by exploiting data obtained from CGM measurements, carbohydrate intake estimates, and insulin infusion recordings. The suggested XGB algorithm might be readily incorporated into existing insulin pumps or deployed as a standalone mobile application.
Tsai et al. [87]	In the present study, researchers used wearable devices to collect PPG signals from nine type 2 diabetic patients to find a correlation between blood glucose levels (BGL) and its collected optical signals. The results of the study showed that 90% accurate glucose predictions can be obtained. To do so, a random forest regression model and an Adaboost model were established.
Aljihmani et al. [88]	Development of a system that recognizes and classifies resting and exertional tasks, and also detects fatigue phases. For this purpose, an analysis based on advanced signal processing and machine learning tools, such as k-nearest neighbors (KNN), decision tree (DT), support vector machine (SVM) and ensemble classifiers (EC), has been applied to identify appropriate models for the classification of rest and effort tasks and the detection of early/late fatigue stages. Training data were obtained from the wrist and finger of the participant’s dominant hand using a 3-axis accelerometer. The ensemble classifier based on the k-NN subspace was considered the best performer in this example with an accuracy of 96.1% in recognizing rest and effort tasks, and ~98% in detecting early and late fatigue stages.
Maritsch et al. [89]	Based on data collected from smartwatch sensors (heart rate variability), this research proposes a machine learning model for detecting hypoglycemia. The classification task of this hypoglycemia alert system is defined as a binary choice between a normal level of blood glucose (negative) and a low blood glucose level (positive). The predictive model used for this task is based on a gradient boosting decision tree (GBDT), with an average accuracy of 82.7%.
Dave et al. [90]:	This study proposes machine learning-based analytical models for probabilistic prediction of hypoglycemia risk in type 1 patients with diabetes. Such systems are designed to be integrated into a smartphone application. The two approaches considered for prediction are logistic regression (LR) and random forests (RF). Indeed, when the time frame is 45 to 60 min, the sensitivity drops from 91% for RF to 58% for LR, giving RF models a considerable advantage over LR models for longer prediction periods.
Stolfi et al. [91]	The objective of this article is to study the different factors that cause the development and occurrence of diabetes. To do this, the authors developed a computer model that summarizes the etiology of the disease and mimics the immunological and metabolic changes associated with it. This method will allow early detection of signs of disease progression, thus providing a tool for self-assessment of people with diabetes. Researchers used 46,170 virtual subjects to develop such a model.
Rghioui et al. [92]	Development of an intelligent system that allows continuous monitoring of the physiological conditions of diabetic individuals and gives doctors the possibility to remotely monitor the health status of these patients, by using sensors integrated in several portable devices (smartphones, smart watches, etc.). This system is able to predict future blood glucose levels, determine the severity of various situations, and classify blood glucose events. In this study, the classification algorithms used were naive Bayes (NB), J48, random tree, ZeroR, SMO (sequential minimal optimization), and OneR. After various tests, the findings reveal that the system based on the J48 algorithm performs excellently, with an accuracy of 99.17%, a sensitivity of 99.47%, and a precision of 99.32%.
Sosale et al. [93]	This article is about a study conducted with 900 participants to evaluate the performance of the Medios artificial intelligence (AI) algorithm in detecting different types of diabetic retinopathy (DR). The technology is a new AI algorithm based on convolutional neural networks using the fundus camera of a smartphone and operating offline. The system shows a high sensitivity (DR: 83.3%; RDR (referable diabetic retinopathy): 93%) and specificity (DR: 95.5%; RDR: 92.5%) for the diagnosis of both referable diabetic retinopathy (RDR) and diabetic retinopathy.

## Data Availability

The study did not report any data.

## Abbreviations

The following abbreviations are used in this manuscript:

IDF	International Diabetes Federation
T1D	Type 1 diabetes
T2D	Type 2 diabetes
GDM	Gestational diabetes
CHD	Coronary heart disease
PAD	Peripheral arterial disease
AI	Artificial intelligence
ML	Machine learning
ECG	Electrocardiography
LapSVM	Laplacian support vector machine
SVM	Support vector machine
DT	Decision Tree
LR	Logistic regression
AdaBoost	Adaptive boosting
URL	Uniform resource locator
MML	Multi-agent machine learning
IP	Internet protocol
ANN	Artificial neural networks
RA	Regression algorithm
SVR	Support vector regression
KNN	K-nearest neighbor
RF	Random forest
Hkmeans	Hierarchical K-means clustering
CGM	Continuous glucose monitoring
PH	Prediction horizons
PSO	Particle swarm optimization
EE	Energy expenditure
DR	Diabetic retinopathy
RDR	Referable diabetic retinopathy
NB	Naive bayes
CNN	Convolutional neural networks
GBDT	Gradient-boosting decision tree
RNN	Recurrent neural network
AP	Artificial pancreas
